# Abdominal perfusion in canine patients with pyometra and sepsis evaluated by Doppler and contrast-enhanced ultrasound

**DOI:** 10.1186/s12917-023-03747-5

**Published:** 2023-09-30

**Authors:** Beatriz Gasser, Ricardo Andres Ramirez Uscategui, Luiz Paulo Nogueira Aires, Diego Iwao Yamada, Priscila Del’Aguila-Silva, Bruna Bressianini Lima, Priscila Silva, Igor Cezar Kniphoff da Cruz, Rafael Kretzer Carneiro, Marcus Antônio Rossi Feliciano

**Affiliations:** 1 Faculdade de Ciências Agrárias e Veterinárias, Universidade Estadual Paulista, Jaboticabal, São Paulo Brasil; 2 Instituto de Ciências Agrárias, Universidade Federal dos Vales do Jequitinhonha e Mucuri, Unaí, Minas Gerais Brasil; 3 Facultad de Medicina Veterinaria y Zootecnia, Universidad del Tolima, Ibagué, Tolima Colombia; 4 Universidade de Marília, Marília, São Paulo, Brasil; 5 Centro de Ciências Rurais, Universidade Federal de Santa Maria, Santa Maria, Rio Grande do Sul Brasil; 6 Universidade do Estado de Santa Catarina, Lages, Santa Catarina, Brasil; 7 Faculdade de Zootecnia e Engenharia de Alimentos, Universidade de São Paulo, Pirassununga, São Paulo Brasil

**Keywords:** Blood flow, Intestinal hemodynamic, CEUS, SIRS, SOFA, Infection

## Abstract

**Background:**

Sepsis is a condition characterized by organic dysfunction, leading to hemodynamic instability and high morbidity and mortality rates in humans and animals. Early identification of perfusion changes and appropriate management of sepsis are crucial for improving patient prognosis. Currently, the Systemic Inflammatory Response Syndrome (SIRS) and Sequential Organ Failure Assessment (SOFA) scores are widely studied for sepsis identification and evaluation of organ dysfunction. However, these scores do not assess gastrointestinal involvement, which is common in this condition. Contrast-enhanced ultrasound (CEUS) and Doppler have been considered promising diagnostic techniques for detecting changes in vascularization and microcirculation in a non-invasive and safe manner, particularly in the gastrointestinal system. This study aimed to evaluate duodenal perfusion using CEUS, as well as abdominal aortic and cranial mesenteric artery blood flow using Doppler ultrasound, and systolic arterial pressure (SAP) in 17 bitches with pyometra and in 10 healthy animals.

**Results:**

The variables were compared between the pyometra and control groups, as well between patients with and without sepsis determined by the SOFA or SIRS scores. Pyometra was found to cause a reduction in abdominal aortic blood flow volume, aortic peak systolic velocity, and resistivity index as evaluated by Doppler ultrasound. Patients with sepsis according to the SOFA criteria only presented lower SAP. In contrast, sepsis animals identified by the SIRS score exhibited lower SAP, aortic peak systolic velocity, aortic blood flow volume, and aortic resistivity index and additionally, higher peak intensity of contrast in the duodenal wall.

**Conclusions:**

Pyometra causes a reduction in abdominal aortic blood flow, which is more pronounced in animals with sepsis identified by the SIRS criteria. These animals also exhibited a decrease in systolic blood pressure and an increase in duodenal perfusion, as evident by CEUS. However, these changes were not observed in patients with sepsis identified by the SOFA criteria. The alterations in intestinal perfusion observed in animals with sepsis indicate the presence of inflammation or dysfunction. In this regard, CEUS proves to be a valuable technique for detecting subtle changes in tissue hemodynamics that may not be apparent in conventional exams.

## Background

Pyometra is characterized by a uterine bacterial infection with the accumulation of inflammatory exudate in the uterine lumen and is one of the most common diseases affecting senior female dogs [1]. The development of pyometra involves hormonal and bacterial factors. During diestrus, there is an increase in uterine secretions, closure of the cervix, and reduction in myometrial contractions under the influence of progesterone [2]. This creates a favorable environment for pregnancy. However, in the absence of an embryo, immunosuppression favors the colonization of bacteria from the vaginal microbiota (predominantly Escherichia coli in 70% of cases) [2, 3]. Additionally, progesterone can lead to pathological proliferation of endometrial glands, intrauterine fluid accumulation, and cyst formation, known as cystic endometrial hyperplasia (CEH), which apparently predisposes to pyometra [1, 2].

Affected dogs with pyometra commonly present with nonspecific signs such as apathy and anorexia, sometimes with a history of reproductive disorders like pseudopregnancy. Clinical examination may reveal vaginal discharges and abdominal palpation of a distended uterus, increasing suspicion of the disease. Blood count and leukogram changes suggestive of acute inflammation also contribute to the diagnosis. When pyometra is suspected, abdominal ultrasonography is indicated to identify uterine distention by anechoic fluid, sometimes with small hyperechoic particles. This can be accompanied by endometrial thickening with cystic areas when associated with CEH [2].

The definitive diagnosis theoretically requires confirmation through cytological analysis of the uterine fluid. Bacterial culture of vaginal discharge is not useful since vaginal bacteria are the main cause of uterine contamination [1]. Pyometra may be associated with a variety of clinical manifestations and has been correlated with sepsis in 60% of affected female dogs [1].

Sepsis is a potentially fatal organ dysfunction caused by a dysregulated immune response to an infection [4]. It can result in complications, mainly due to poor tissue perfusion. Hemodynamic instability and an uncontrolled inflammatory response resulting from sepsis can lead to the development of multiple organ dysfunction syndrome (MODS) in approximately 50% of dogs, increasing the mortality rate from 25 to 75% [5]. Among the MODS documented in humans, gastrointestinal dysfunction was reported in 59% of cases [6].

Gastrointestinal dysfunction can cause hyporexia or anorexia, an inability to tolerate enteral feedings, decreased intestinal motility, hemorrhagic diarrhea, increased intestinal permeability, and bacterial translocation [7]. Sepsis can lead to intestinal barrier dysfunction, triggering bacterial translocation and significantly worsening the clinical status, while also promoting changes in intestinal motility and nutrient absorption [7, 8]. The incidence of gastrointestinal dysfunction in infected animals is not well-documented due to the difficulties in assessment; however, it is considered common [7]. Furthermore, the lack of valid biomarkers for gastrointestinal dysfunction makes it challenging to diagnose alterations in patients with infections.

Early identification of organ dysfunctions and proper management of sepsis in the initial hours following its development can significantly improve patient prognosis. Therefore, sepsis screening systems need to exhibit high accuracy to allow for timely and appropriate treatment. Various criteria have been proposed for sepsis screening, with the Systemic Inflammatory Response Syndrome (SIRS) score and the Sequential Organ Failure Assessment (SOFA) being the most extensively studied [4, 9].

Currently, the SIRS score serves as the standard method for determining sepsis in dogs. It evaluates the inflammatory response to infection by considering alterations in rectal temperature (< 38.1°C or > 39.2°C), heart rate (> 120 bpm), respiratory rate (> 20 rpm), and leukocyte count (< 6 or > 16 × 10^3^/µL and/or bands > 3%) as identification criteria. Animals presenting two or more of these alterations, along with a suspected or confirmed infection, are considered to have sepsis. While this score exhibits high sensitivity (97%), it displays low specificity (64%), resulting in a high rate of false positives [10].

In humans, the SOFA score was introduced to identify common organ system dysfunctions in sepsis, such as respiratory dysfunction indicated by a low ratio of arterial oxygen pressure to inspired oxygen fraction (PaO_2_/FiO_2_), coagulation dysfunction manifested as thrombocytopenia, hepatic dysfunction indicated by hyperbilirubinemia, renal dysfunction indicated by an increase in serum creatinine, cardiovascular dysfunction manifested as hypotension, and neurological dysfunction indicated by a decrease in the Glasgow Coma Scale. The SOFA score evaluates the presence of sepsis and the severity of the patient based on the degree of organ dysfunction. Individuals with two or more points in this score, along with a suspected or confirmed infection, are diagnosed with sepsis [9]. Although this score has been evaluated in dogs and considered a useful prognostic indicator in the intensive care unit [11], its accuracy for identifying or staging sepsis in animals has not yet been assessed.

It is important to note that neither of these screening systems assesses gastrointestinal function for sepsis identification or staging. Considering the importance of intestinal function and the challenges in identifying alterations in this system, the evaluation of intestinal perfusion using contrast-enhanced ultrasound (CEUS) has been considered a promising diagnostic technique. CEUS enables the detection of changes in vascularization and microcirculation that are imperceptible with other methods, in a non-invasive and safe manner [12]. This technique has already been described for detecting changes in renal perfusion in dogs with pyometra and sepsis-related acute kidney injury [13]. Preliminary studies have also described its accuracy in identifying intestinal disorders in humans and dogs [14, 15], but no studies have evaluated intestinal perfusion in patients with infection.

Given this issue, the objective of this study was to evaluate duodenal perfusion, blood flow in the abdominal aorta and cranial mesenteric artery (CMA) using CEUS and Doppler techniques, as well as systolic arterial pressure (SAP), in healthy female dogs and female dogs with pyometra, with or without sepsis as determined by SIRS or SOFA criteria.

## Methods

The sample size for this study was calculated using G*Power® software (Version, Universität Kiel, Germany). A total of 20 bitches with pyometra and 10 healthy bitches were included, aiming for a significance level of 1% (α = 0.01) and a statistical power of 70% (1—β = 0.70), based on the results of a study that evaluated organ perfusion with CEUS in septic pigs [16].

All animals were treated at the Obstetrics and Animal Reproduction sector of the Veterinary Hospital "Governador Laudo Natel" of FCAV-UNESP in Jaboticabal, SP, Brazil, and were included in the study with the voluntary authorization of their owners for surgical sterilization.

A total of 27 bitches were included in the analysis: 10 healthy adult bitches formed the control group, and 17 bitches had pyometra (3 bitches from the pyometra group were excluded for not meeting the inclusion criteria). The control group consisted of 5 Shih Tzu, 1 Border Collie, 1 Bull Terrier, 1 Dachshund, 1 Lhasa Apso, and 1 Mongrel, ranging in age from 1 to 9 years old (mean ± SD: 6.1 ± 2.4 years) and weighing 4 to 22 kg (9.4 ± 5.9 kg). The pyometra group consisted of 6 Mongrels, 5 Shih Tzu, 2 Labrador Retrievers, 1 Pinscher, 1 Pitbull Terrier, 1 Poodle, and 1 Pug, ranging in age from 3 to 12 years old (7.6 ± 2.6 years) and weighing 4 to 38 kg (15.7 ± 13.2 kg).

Before the experiment, all patients in both groups underwent physical examination, laboratory tests, and ultrasound examination. In the control group, the laboratory test results were used to confirm the health of the patients before the study. For animals suspected of having pyometra, abdominal ultrasound was performed to pre-diagnose the condition before their inclusion in the research. The definitive diagnosis of pyometra was confirmed by cytological analysis (Gram stain) of the uterine contents obtained through sterile aspiration after ovariohysterectomy.

At physical examination, measurements were taken for heart rate (HR), respiratory rate (RR), rectal temperature (T°C), degree of dehydration and level of consciousness using the modified Glasgow Coma Scale for dogs [17]. Additionally, Systolic arterial pressure (SAP) was measured using the indirect vascular Doppler method. The region of the median or digital palmar artery of one of the thoracic limbs was shaved, ultrasound gel was applied and a vascular Doppler sensor was used to seek arterial flow sound. An inflatable cuff (with approximately 40% of the limb's circumference) was placed proximal to the sensor and connected to an analog sphygmomanometer, the cuff was then inflated and deflated to identify the SAP, as the point at which the sound of arterial flow returns and measured in triplicate [18].

Venous and arterial blood samples were collected immediately for laboratory tests, including blood gas analysis, complete blood count, serum creatinine concentration (mg/dL), and total bilirubin concentration (mg/dL). These parameters were used to classify animals as septic or non-septic based on the SIRS or SOFA criteria [10, 11].

After blood samples, a wide ventral abdominal shaving was performed and the patients received an intravenous catheter (22G) in the left cephalic vein. They were then positioned for ultrasound evaluation. During the ultrasound examination, the dogs were physically restrained in the supine position on a padded triangular support, with their head facing the monitor and their body parallel to the ultrasound device. Before the examination, a specific gel was applied to improve the contact between the transducer and the skin.

The ultrasound examinations were conducted using an ACUSON S2000 device (Siemens Company, Munich, Germany) equipped with a linear multifrequency matrix transducer ranging from 4 to 9 MHz. In the pyometra group, measurements were taken for uterine diameter, wall thickness, and luminal thickness at the largest portion of the uterus. In the control group, the uterus and ovaries were evaluated to confirm a normal ultrasonographic appearance [19].

The abdominal ultrasound examination in B-mode began with the evaluation of a segment of the descending duodenum in the longitudinal section. The presence or absence of motility was assessed, and the wall thickness was measured [20].

Next, the cranial mesenteric artery (CMA) and abdominal aorta were located using the color Doppler mode. Color mapping was then performed to measure the middle area of each artery in cross-section during diastole and systole. These measurements were taken in triplicate [21]. The cranial mesenteric artery measurements were obtained near its origin, while those of the abdominal aorta were acquired in the portion caudal to the left kidney.

Pulsed Doppler was activated, and the cursor was positioned in the central portion of each artery, ensuring that the axis of the sound beam and the axis of the vessel were parallel to each other with an angle of insonation below 60°. The sample volume was set to measure 2/3 the diameter of the vessel in order to obtain clean flowmetric tracings. Once a clear tracing was obtained, the image was frozen, and the following parameters were collected automatically by the equipment: peak systolic velocity (PS), end-diastolic velocity (ED), time-averaged maximum velocity (TaMax), time-averaged minimum velocity (TaMin), resistivity index (RI), and pulsatility index (PI).

Subsequently, the blood flow volumes (BFV ml/min/kg) of the cranial mesenteric artery and abdominal aorta were calculated using the mean velocity ($Vmean=\frac{PS+ED}{2}$; cm/s), mean vessel area (A cm^2^), and the weight of the animal (W kg) according to the following formula [21]:

$$BFV=\frac{Vmean\times60\times A}W.$$

After completing the Doppler examination, contrast-enhanced ultrasound (CEUS) was performed using harmonic imaging software (Cadence, Siemens Company, Munich, Germany). The ultrasound image of a duodenal portion was centered on the screen, aiming to obtain a clear and isolated image in the longitudinal section of the duodenum. The acoustic power (MI) was set at 0.07, and parameters such as gain, depth, dynamic range, frequency, and focus were optimized during the initial evaluation to ensure excellent image quality. These settings were kept constant throughout the experiment.

The ultrasound contrast agent SonoVue® (Bracco, São Paulo, Brazil) was then administered as a bolus intravenously at a dose of 0.01 mL/kg using a 1 mL syringe, followed by a 5 mL saline flush. The contrast dose used was the same as described in a previous study on renal perfusion in dogs with pyometra [13], and it was validated through pilot studies in healthy animals and animals with pyometra to ensure optimal contrast enhancement of intestinal tissues. The time of contrast application was considered as T0, and the recording of the dynamic ultrasound assessment of the duodenum was performed in video format for a duration of 180 s for further analysis, as described and validated in the literature [15, 22].

**Fig. 1:**
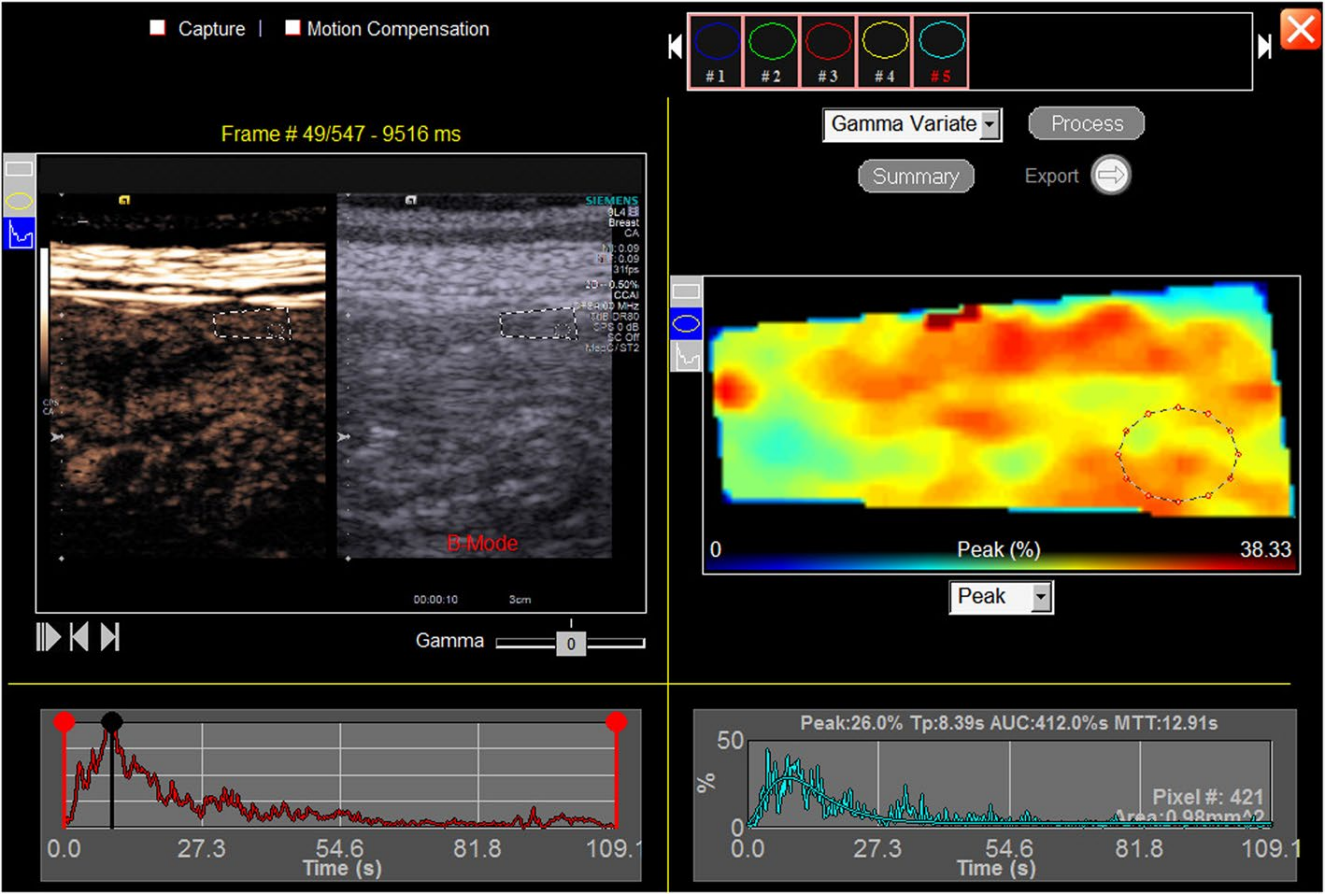
A representative contrast-enhanced ultrasound (CEUS) image of a portion of the duodenum in longitudinal section with the area of interest defined (left image—dashed line) including the largest area of its wall, with one of the 5 regions of interest (ROI) defined inside (right image—dashed circle with dots), with approximately 1 mm^2^. The corresponding time-intensity curve of each area is shown below each image, represented by red and cyan lines

Once the acquisition was completed, all saved videos were transferred to an offline analysis module (DICOM® Digital Imaging and Communications in Medicine, Rosslyn, VA, USA). In the software the evaluator selected a specific area of interest (ROI) within the duodenal region, this ROI is automatically converted to a colored map. In this map the evaluator selected five circular ROIs of approximately 1 mm^2^, excluding regions with "vessel characteristics" (areas with intense red coloring). The software automatically mounted time-intensity curves (TIC) that are representative of the selected area (Fig. 1) and calculated perfusion parameters, including the number of pixels (Pixel), peak intensity (Peak in %), time to peak intensity (Tp in s), mean transmission time (MTT in s), and area under the curve (AUC) [13].

The software's motion compensation tool was not applied in this study as it interfered with the generation of the TIC, once the duodenum has natural movements. If the evaluation was compromised by patient or duodenal movement, the examination was repeated until a reliable collection was obtained for the generation of an appropriate TIC.

The peak of intensity in % corresponds to the percentage of the contrast filled out of the total pixels in the evaluated ROI, and there may be variation in the total number of pixels depending on the size of the image of the target organ during ultrasound evaluation. Therefore, this value was also converted to peak intensity in pixels (Peak pixel) using the formula:

$$Peak\;pixel\;=\frac{Peak\;\%\;\times\;Pixels}{100}.$$

The statistical analysis of all these variables was performed using the R software (R Foundation for Statistical Computing, Austria). The variables were compared between the pyometra group and the control group, as well as among patients classified as sepsis or not by the SOFA and SIRS scores. The Kruskal–Wallis test was used for the comparisons, and the results are presented as the median ± interquartile range (IQR). Statistical significance was set at *p* < 0.05.

## Results

Of the 17 animals with pyometra, 14 (82.35%) had genital discharges. The reported clinical signs by the owners were apathy and anorexia for a period of up to one week before the appointment. The mean diameter, wall thickness, and luminal thickness of the uterus in the pyometra group were 2.19 ± 1.40 cm, 0.36 ± 0.15 cm, and 1.64 ± 1.47 cm, respectively. No animal presented any comorbidities in both groups. The macroscopic appearance of the uterine fluid was purulent in 12 out of 17 animals and bloody in 5 out of 17. Gram staining of the uterine fluid revealed exudate in all animals. In 8 out of 17 animals, the bacterial type could not be identified due to the high number of inflammatory cells. In 7 out of 17 animals, mostly Gram-negative bacilli were observed, while in 2 out of 17 animals, mostly Gram-positive cocci were present.

When comparing the control group (*n* = 10) with the pyometra group (*n* = 17), the pyometra group showed lower values of aortic blood flow volume (*p* = 0.001), aortic peak systolic velocity (*p* = 0.006), and aortic resistivity index (*p* = 0.042) as evaluated by Doppler ultrasound. The other clinical, B-mode, and CEUS variables were similar between the two groups (Table 1).

**Table 1:** Median ± IQR of aortic, cranial mesenteric artery (CMA), and duodenal perfusion in healthy and pyometra bitches

Variables	Control	Pyometra	*P*-value
**SAP (mmHg)**	176 ± 35.8	178 ± 45.3	0.342
**B-mode**			
**Duodenal thickness (cm)**	0.39 ± 0.12	0.35 ± 0.11	0.632
**Aorta Doppler**			
**BFVaorta (ml/min/kg)**	132 ± 96.5	98.1 ± 28.6	0.001*
**PSaorta (cm/s)**	117 ± 22,5	90.7 ± 22.5	0.006*
**RIaorta**	0.9 ± 0,05	0.84 ± 0.07	0.042*
**PIaorta**	3.64 ± 3,83	2.86 ± 1.63	0.209
**TaMinaorta (cm/s)**	15.1 ± 6,91	13.1 ± 5.75	0.436
**EDaorta (cm/s)**	12.4 ± 8,05	14.4 ± 8.38	0.547
**TaMaxaorta (cm/s)**	30.1 ± 13,5	27.6 ± 12.2	0.620
**Cranial Mesenteric Artery Doppler**			
**BFVcma (ml/min/kg)**	44.6 ± 16.6	42,2 ± 27.4	0.798
**PScma (cm/s)**	105 ± 30.9	115 ± 41.8	0.551
**RIcma**	0.83 ± 0.03	0.81 ± 0.05	0.388
**PIcma**	3.12 ± 1.6	2.54 ± 0.71	0.202
**TaMincma (cm/s)**	16.8 ± 8.68	19.2 ± 8.03	0.475
**EDcma (cm/s)**	17.6 ± 6.22	20.6 ± 8.45	0.344
**TaMaxcma (cm/s)**	33 ± 14.9	39.3 ± 16.3	0.328
**CEUS**			
**PeakDuodenum (Pixel)**	28.9 ± 39.4	42.2 ± 56.9	0.175
**PeakDuodenum (%)**	11.2 ± 9.29	19.9 ± 12.3	0.132
**AUCDuodenum (%s)**	271 ± 279	432 ± 777	0.340
**TpDuodenum (s)**	11.6 ± 16.6	11.5 ± 21.4	0.422
**MTTDuodenum (s)**	23.9 ± 24.9	19.6 ± 32.5	0.841
**PixelDuodenum**	361 ± 227	408 ± 240	0.980

*SAP* Systolic Arterial Pressure, *BFV* Blood Flow Volume, *aorta* abdominal aorta artery, *cma* cranial mesenteric artery, *PS* peak systolic velocity, *RI* resistivity index, *PI* pulsatility index, *ED* end diastolic velocity, *TaMin* time-averaged minimum velocity, *TaMax* time-averaged maximum velocity, *Peak* peak intensity of contrast, *AUC* area under the curve, *Tp* time to peak intensity, *MTT* mean transit time

^*^Considered statistically significant by t-Student test *p* ≤ 0.05

When the SOFA score was applied to identify "sepsis", only 4 out of 27 animals (24%) had a SOFA score greater than 2 and were diagnosed with sepsis according to this score, all belonging to the pyometra group and exhibited dysfunction in the following systems: 3 out of 27 (11%) had renal alterations (elevated creatinine), 3 out of 27 (11%) had thrombocytopenia, 1 out of 27 (4%) had respiratory dysfunction (lower PaO_2_/FiO_2_), and 1 out of 27 (4%) had a change in the level of consciousness. No hepatic dysfunction or hypotension was observed based on the SOFA criteria.

When comparing patients with sepsis according to the SOFA score to patients without sepsis, only systolic arterial pressure (SAP) was significantly lower in the sepsis animals (*p* = 0.005). The other variables did not show significant differences (Table 2).

**Table 2:** Median ± IQR of aorta, cranial mesenteric artery (CMA) and duodenal perfusion of sepsis and not-sepsis bitches classified by SOFA score

Variables	Not-sepsis	Sepsis SOFA	*P*-value
**SAP (mmHg)**	187 ± 35.3	120 ± 23.9	0.005*
**B-Mode**			
**Duodenal thickness (cm)**	0.39 ± 0.09	0.37 ± 0.19	0.732
**Aorta Doppler**			
**BFVaorta (ml/min/kg)**	109 ± 40.6	114 ± 82	0.838
**PSaorta (cm/s)**	103 ± 25.6	85.5 ± 23.6	0.280
**RIaorta**	0.87 ± 0.06	0.83 ± 0.08	0.408
**PIaorta**	3.07 ± 2.32	2.79 ± 2.04	0.585
**TaMinaorta (cm/s)**	14.1 ± 6.45	12.6 ± 4.43	0.672
**EDaorta (cm/s)**	13.7 ± 8.45	13.8 ± 7.3	0.976
**TaMaxaorta (cm/s)**	28.9 ± 13.2	26.3 ± 8.64	0.709
**Cranial Mesenteric Artery Doppler**			
**BFVcma (ml/min/kg)**	39 ± 39.3	42.8 ± 26.9	0.747
**PScma (cm/s)**	109 ± 74.1	113 ± 41.7	0.816
**RIcma**	0.82 ± 0.06	0.81 ± 0.08	0.550
**PIcma**	2.32 ± 1.26	2.46 ± 0.59	0.550
**TaMincma (cm/s)**	15.3 ± 12.7	20.4 ± 8.4	0.739
**EDcma (cm/s)**	16.3 ± 11.5	21.8 ± 5.85	0.600
**TaMaxcma (cm/s)**	31.9 ± 30.7	42.4 ± 14.4	0.786
**CEUS**			
**PeakDuodenum (Pixel)**	34.9 ± 44.5	71.8 ± 51.5	0.375
**PeakDuodenum (%)**	14.8 ± 12.6	18.7 ± 16.9	0.539
**AUCDuodenum (%s)**	299 ± 302	321 ± 950	0.733
**TpDuodenum (s)**	11.5 ± 22.1	10.9 ± 5.62	0.580
**MTTDuodenum (s)**	23.8 ± 37.1	18.9 ± 11.9	0.453
**PixelDuodenum**	409 ± 240	321 ± 224	0.838

*SAP* Systolic Arterial Pressure, *BFV* Blood Flow Volume, *aorta* abdominal aorta artery, *cma* cranial mesenteric artery, *PS* peak systolic velocity, *RI* resistivity index, *PI* pulsatility index, *ED* end diastolic velocity, *TaMin* time-averaged minimum velocity, *TaMax* time-averaged maximum velocity, *Peak* peak intensity of contrast, *AUC* area under the curve, *Tp* time to peak intensity, *MTT* mean transit time

^*^Considered statistically significant by t-Student test *p* ≤ 0.05

When SIRS score was applied to identify “sepsis”, of the 27 animals, 15 were considered in sepsis (55.50%), all belonging to the pyometra group, although 9 animals in the control group had a score ≥ 2, did not show signs of infection and therefore were not considered in sepsis. Of the control group, elevation of RR was the most frequent alteration in 9/10 animals (90%), followed by elevated of HR in 7/10 animals (70%), rectal temperature was elevated in 3/10 (30%) animals and leukopenia was observed in 1/10 (10%). In pyometra group, all animals 17/17 (100%) presented elevated RR, 11/17 (65%) had altered leukocytes, 10 leukocytosis and 1 leukopenia, 10/17 (59%) presented elevated HR and 6/17 (35%) altered rectal temperature, 4 with T < 38.0°C and 2 with T > 39.2°C.

Comparing sepsis and not-sepsis animals identified by SIRS score, sepsis animals showed lower systolic blood pressure (*p* = 0.042), aortic peak systolic velocity (*p* = 0.009), aortic blood flow volume (*p* = 0.014) and aortic resistivity index (*p* = 0.038), whereas, CEUS evaluation revealed that the peak intensity of contrast in pixels of duodenal parenchyma was higher (*p* = 0.049). The other variables did not differ between groups (Table 3).

**Table 3:** Median ± IQR of aorta, cranial mesenteric artery (CMA) and duodenal perfusion of sepsis and not-sepsis bitches classified by SIRS score

Variables	Not-sepsis	Sepsis SIRS	*P*-value
**SAP (mmHg)**	195 ± 25.3	170 ± 45.1	0.042*
**B-Mode**			
**Duodenal thickness (cm)**	0.39 ± 0.09	0.35 ± 0.12	0.524
**Aorta Doppler**			
**BFVaorta (ml/min/kg)**	148 ± 54.6	99.9 ± 26.1	0.014*
**PSaorta (cm/s)**	115 ± 22.9	89.5 ± 22.6	0.009*
**RIaorta**	0.89 ± 0.05	0.84 ± 0.07	0.038*
**PIaorta**	4.00 ± 1.68	3.27 ± 1.84	0.294
**TaMinaorta (cm/s)**	14.9 ± 6.52	13 ± 5.92	0.448
**EDaorta (cm/s)**	12.7 ± 7.57	14.5 ± 8.78	0.562
**TaMaxaorta (cm/s)**	30 ± 12.9	27.4 ± 12.4	0.599
**Cranial Mesenteric Artery Doppler**			
**VFScma (ml/min/kg)**	43.8 ± 15.2	42.5 ± 29.3	0.881
**PScma (cm/s)**	109 ± 29.1	113 ± 44.4	0.742
**RIcma**	0.83 ± 0.03	0.81 ± 0.05	0.206
**PIcma**	3.05 ± 1.47	2.51 ± 0.73	0.263
**TaMincma (cm/s)**	17.3 ± 8.04	19.1 ± 8.51	0.578
**EDcma (cm/s)**	18.1 ± 5.93	20.7 ± 8.91	0.366
**TaMaxcma (cm/s)**	34.2 ± 14.2	39.3 ± 17.2	0.411
**CEUS**			
**PeakDuodenum (Pixel)**	28.9 ± 33.9	63.9 ± 58.6	0.049*
**PeakDuodenum (%)**	11.2 ± 12.5	19.9 ± 11.6	0.143
**AUCDuodenum (%s)**	288 ± 289	350 ± 408	0.884
**TpDuodenum (s)**	13.5 ± 21.6	10.8 ± 5.65	0.770
**MTTDuodenum (s)**	27.9 ± 39.9	18.2 ± 13.1	0.188
**PixelDuodenum**	361 ± 269	408 ± 240	0.981

*SAP* Systolic Arterial Pressure, *BFV* Blood Flow Volume, *aorta* abdominal aorta artery, *cma* cranial mesenteric artery, *PS* peak systolic velocity, *RI* resistivity index, *PI* pulsatility index, *ED* end diastolic velocity, *TaMin* time-averaged minimum velocity, *TaMax* time-averaged maximum velocity, *Peak* peak intensity of contrast, *AUC* area under the curve, *Tp* time to peak intensity, *MTT* mean transit time

^*^Considered statistically significant by t-Student test *p* ≤ 0.05

## Discussion

The diagnosis of pyometra was based on clinical, ultrasonographic, and cytological characteristics of the disease. Normal uterus ultrasound characteristics in bitches during early diestrus include a diameter of 1.31 ± 0.09 cm without anechoic fluid in the lumen [19]. In our study, we observed a uterine diameter of 2.19 ± 1.40 cm with intraluminal thickness of 1.64 ± 1.47 cm filled with echoic and anechoic fluid, which is consistent with pyometra. The cytological examination of uterine fluid in pyometra cases typically reveals a large number of neutrophils and the presence of intra- and/or extracellular bacteria [23], which also corroborates our findings of inflammatory cells, Gram-negative bacilli, and Gram-positive cocci in the cytological exam of bitches with pyometra.

When comparing the variables studied between animals with pyometra and healthy animals, we observed lower values in bitches with pyometra for aortic blood flow volume, aortic peak systolic velocity, and aortic resistivity index. Similarly, in the comparison of animals with sepsis according to the SIRS criteria to those without sepsis, sepsis animals showed lower values for systolic blood pressure, aortic peak systolic velocity, aortic blood flow volume, and aortic resistivity index ad additionally, sepsis animals exhibited a higher peak intensity of contrast on the duodenal wall. However, when comparing animals with sepsis according to the SOFA criteria to those without sepsis, only systolic blood pressure was found to be lower in animals with sepsis.

Overall, these findings highlight the presence of cardiovascular alterations in bitches with pyometra and sepsis. The differences observed between the different groups and criteria used for sepsis diagnosis suggest that the severity and extent of these alterations may vary depending on the specific condition and diagnostic criteria employed.

One of the main systemic and clinically applicable results to discuss is the statistical reduction of systolic arterial pressure (SAP) in animals with sepsis, even though it remained within the reference range (SAP < 90mmHg is considered hypotension in dogs) [24]. This finding is consistent with a study in rats with induced sepsis, which showed that SAP did not differ in the first 2 days but significantly decreased after that period [25]. These findings may explain the absence of decreased blood pressure in animals with pyometra but its presence in animals with sepsis, which may be related to the time of evolution and escalation of this syndrome.

Arterial hypotension, considered one of the main criteria for evaluating human sepsis, does not appear to be as constant in dogs. A retrospective study evaluating 144 dogs with sepsis found that 17.5% of them expressed hypotension requiring treatment, ranking fourth among observed organ dysfunctions after coagulation, hepatic, and respiratory dysfunction [5]. In a previous study by our research group that evaluated 20 bitches with pyometra and acute kidney injury, no hypotension was detected, and the mean SAP was 115 ± 32.5 mmHg [13].

In this context, it is known that vasodilation is one of the main cardiovascular alterations triggered by sepsis, leading to a reduction in blood flow volume and the aortic resistivity index, as observed in bitches with pyometra and more markedly in those with sepsis identified by the SIRS criteria [26]. The drop in vascular resistance was evidenced by the reduction in aortic resistivity index, systolic velocity, and blood flow volume in bitches with pyometra and more markedly in those with sepsis. This change in aortic blood flow resulted in a significant decrease in systolic blood pressure (without reaching hypotension) only in animals with sepsis, indicating the loss of vasomotor tone in the central resistance vessels and the ability to maintain mechanisms regulating peripheral blood pressure in dogs.

Doppler vascular parameters of the aorta artery in rabbits with induced sepsis showed changes in resistance and velocity within the first 3–4 h, which then returned to normal, indicating an effective compensatory mechanism to maintain tissue perfusion [27]. The absence of alterations in cranial mesenteric artery Doppler parameters observed in our study may be related to the time of evolution of pyometra since the infection in these animals was spontaneous, and the interval between infection development and seeking veterinary care usually takes several days. It is worth mentioning that the cranial mesenteric artery Doppler values found in healthy animals in our study are consistent with the literature for the species, as well as the abdominal aorta variables [28]. However, we did not find any studies comparing these parameters in dogs with infections to compare with our findings.

In patients with pyometra, we identified a reduction in aortic blood flow that was not reflected in a reduction of cranial mesenteric blood flow. When we examined duodenal perfusion using CEUS, we found that this function was also maintained without reflecting the change in systemic blood flow. Similar discrepancies were observed in septic rats [29], where duodenal perfusion tended to increase while ileum and colon blood flow decreased. Increase in jejunal flow was described in pigs in septic shock [30], indicating that microcirculatory flow in this tissue was actively maintained by local mechanisms during sepsis, while gastric and colonic flow were reduced. The heterogeneity in the hemodynamic response of different intestinal regions to infection emphasizes the importance of specific studies for each region.

A study evaluating blood flow in the small intestine of rats with induced peritonitis using radioactive microspheres for four days found an increase in septic animals compared to controls [25]. These findings were reproduced in our study using CEUS, which does not involve radioactivity or invasiveness, and may be related to another study in dogs with inflammatory bowel disease that discovered an increase in contrast enhancement peak and area under the curve during CEUS evaluation [15]. It is possible to infer that the change in intestinal flow in patients with sepsis is due to inflammation or dysfunction of the organ. When the flow is above the values found in healthy or pyometra patients, it is no longer possible to consider it a physiological compensation.

Regarding the parameters used to assess intestinal functionality, the B-mode duodenal wall thickness was similar in healthy, pyometra, and sepsis patients. A study conducted in dogs demonstrated that duodenal thickness did not differ between normal animals and dogs with inflammatory bowel disease [20], which is consistent with our results.

Finally, this study has some limitations, which will be discussed here. During the CEUS exam, we encountered difficulties in obtaining high-quality images due to the challenge of stabilizing the target organ within the sonographic beam for a continuous period of 3 min, while the intestinal loops exhibit peristalsis and movement. These difficulties are reflected by the high interquartile range values of the CEUS parameters, which is a sensitive statistical measure of dispersion. Previous literature has described variations in the blood flow of the cranial mesenteric artery in dogs during the pre and postprandial periods, with a significant decrease in the resistivity index (RI) and pulsatility index (PI), and an increase in mean velocity, flow volume, and end-diastolic velocity during the postprandial period [28]. In our study, we did not control or evaluate the effects of food and nutritional status due to the clinical characteristics of the spontaneous model of abdominal infection and sepsis used. However, according to the owners, the dogs in the present study experienced anorexia for up to one week before their appointment.

In sepsis studies, animals are generally induced to develop sepsis. In our study, some dogs with pyometra were in a sepsis state while others were not. This aspect makes the analysis more challenging as it practically forms three groups: healthy, infected, and septic. The SOFA criteria, which are commonly used to identify sepsis, do not allow the identification of sepsis in animals that apparently exhibit significant alterations in systemic and tissue hemodynamics as identified by the SIRS criteria. This limitation leads to low accuracy in diagnosing sepsis, potentially resulting in delayed treatment and poor outcomes. Additionally, it compromises the statistical comparisons that can be made among the groups.

## Conclusions

Pyometra causes a reduction in abdominal aortic blood flow, which is more pronounced in animals with sepsis identified by the SIRS criteria. These animals also exhibited a decrease in systolic blood pressure and an increase in duodenal perfusion, as evident by CEUS. However, these changes were not observed in patients with sepsis identified by the SOFA criteria. The alterations in intestinal perfusion observed in animals with sepsis indicate the presence of inflammation or dysfunction. In this regard, CEUS proves to be a valuable technique for detecting subtle changes in tissue hemodynamics that may not be apparent in conventional exams.

## Data Availability

The datasets generated during and/or analyzed during the current study are available from the corresponding author on reasonable request.

## Abbreviations

SIRS: Systemic Inflammatory Response Syndrome
SOFA: Sequential Organ Failure Assessment
CEUS: Contrast-enhanced ultrasound
CMA: Cranial mesenteric artery
SAP: Systolic Arterial Pressure
MODS: Multiorgan dysfunction syndrome
HR: Heart rate
RR: Respiratory rate
T: Rectal temperature
PS: Peak systolic velocity
ED: End-diastolic velocity
TaMax: Time-averaged maximum velocity
TaMin: Time-averaged minimum velocity
RI: Resistivity index
PI: Pulsatility index
BFV: Blood flow volume
ROI: Regions of interest
TIC: Time-intensity curve
Tp: Time to peak intensity
MTT: Mean transmission time
AUC: Area under the curve
IQR: Interquartile range
